# A Dynamic Response Regulator Protein Modulates G-Protein–Dependent Polarity in the Bacterium *Myxococcus xanthus*

**DOI:** 10.1371/journal.pgen.1002872

**Published:** 2012-08-16

**Authors:** Yong Zhang, Mathilde Guzzo, Adrien Ducret, Yue-Zhong Li, Tâm Mignot

**Affiliations:** 1 Laboratoire de Chimie Bactérienne, CNRS UPR9043, Institut de Microbiologie de la Méditerranée, Université Aix-Marseille, Marseille, France; 2 State Key Laboratory of Microbial Technology, College of Life Science, Shandong University, Jinan, China; University of Geneva Medical School, Switzerland

## Abstract

Migrating cells employ sophisticated signal transduction systems to respond to their environment and polarize towards attractant sources. Bacterial cells also regulate their polarity dynamically to reverse their direction of movement. In *Myxococcus xanthus*, a GTP-bound Ras-like G-protein, MglA, activates the motility machineries at the leading cell pole. Reversals are provoked by pole-to-pole switching of MglA, which is under the control of a chemosensory-like signal transduction cascade (Frz). It was previously known that the asymmetric localization of MglA at one cell pole is regulated by MglB, a GTPase Activating Protein (GAP). In this process, MglB specifically localizes at the opposite lagging cell pole and blocks MglA localization at that pole. However, how MglA is targeted to the leading pole and how Frz activity switches the localizations of MglA and MglB synchronously remained unknown. Here, we show that MglA requires RomR, a previously known response regulator protein, to localize to the leading cell pole efficiently. Specifically, RomR-MglA and RomR-MglB complexes are formed and act complementarily to establish the polarity axis, segregating MglA and MglB to opposite cell poles. Finally, we present evidence that Frz signaling may regulate MglA localization through RomR, suggesting that RomR constitutes a link between the Frz-signaling and MglAB polarity modules. Thus, in *Myxococcus xanthus*, a response regulator protein governs the localization of a small G-protein, adding further insight to the polarization mechanism and suggesting that motility regulation evolved by recruiting and combining existing signaling modules of diverse origins.

## Author Summary

Migrating cells have evolved a molecular compass to rapidly respond to environmental signals. During chemotaxis, small G-proteins and their regulators are activated and determine a leading cell edge towards attractant molecules. Bacteria also move across surfaces in a directed manner. The rod-shaped bacterium *Myxococcus xanthus* can switch its direction of movement in a process where the cell poles exchange roles (reversal), allowing complex multicellular behaviors. In *Myxococcus*, a small G-protein, MglA, determines the leading cell pole. In this study, we investigated how MglA localizes to the pole and found that its localization depends on the dual complementary action of RomR and MglB. In this process, RomR targets MglA to the pole while, in turn, MglB prevents its accumulation at the back of the cell. Moreover, RomR potentially links MglA to the Frz signal transduction pathway, a chemosensory system controlling the reversal frequency. The results provide a new molecular basis to understand motility regulation in a bacterium, which may have arisen from co-optation and branching of prokaryotic and eukaryotic-like signaling modules.

## Introduction

In living organisms, cell polarization underlies many developmental and cellular processes, such as budding in yeast, cell migration and bacterial differentiation [1]–[3]. In eukaryotic cells, polarization mechanisms ensure the asymmetric positioning of subcellular organelles and its transmission upon cell division [4]. Due to their small sizes bacterial cells have long been thought to be unorganized compartments, proven a misconception with the discovery that bacterial cells also contain subcellular structures and micro-compartments [5], [6]. In particular, the bacterial cell pole orchestrates many processes, for example chemotaxis, flagellum assembly and even chromosome segregation [5], [7]–[10]. Polar targeting often resides on scaffolding proteins or complexes targeted to the pole through several possible mechanisms: interaction with the forming division septum [7], [8], recognition of specific lipid polar microdomains [11], [12] and even direct geometric recognition of polar curvature [13], [14]. In some cases polar localization must be dynamically regulated to segregate cell division inhibitors [9], [10], degrade a cell cycle regulator [15], or invert the direction of cell movement [16], [17]. In this study, we identify a regulator directing dynamic pole-specific activation of motility complexes in the bacterium *Myxococcus xanthus*.

In *Myxococcus xanthus*, a rod-shaped delta-proteobacterium, two distinct macromolecular machines drive motility. The first system, a type-IV pilus (T4P) engine located at the leading cell pole binds to neighboring cells or the extracellular matrix to pull the cells forward by retraction (S-motility, [18]). The second system, the recently characterized Agl-Glt complex, is also assembled at the leading cell pole where it forms focal adhesion-like complexes which distribute along the cell body to propel the cell (A-motility, [19]–[21]). *Myxococcus* cells direct their motility by changing their direction of movement periodically in a process where the poles exchange roles, allowing the cells to resume movement in the opposite direction. These reversals require switching the directionality of the A- and S- molecular engines synchronously. Recent cytological experiments have suggested that pre-assembled T4P exist at both cell poles but are only active at one cell pole, due to asymmetric pilus-associated proteins like FrzS, Tgl, PilB and PilT [22]–[24]. The switching of pilus directionality would thus occur following pole-to-pole switching of these factors, which has been experimentally observed for FrzS and PilT [22], [24]. The mechanism allowing the directional switch of the A-motility system is less clear but seems to involve the synchronous pole-to-pole switching of essential Agl-Glt complex proteins, which has been shown for AglZ and AglQ [21], [25].

What drives the dynamic behavior of the A (AglZ, AglQ) and S motility proteins (FrzS, PilT) during reversals? Recent studies identified the MglA and MglB proteins as central regulators of the reversal cycle. MglA is the founding member of a group of bacterial G-proteins of the Ras superfamily [16], [17], [26]. As for all other Ras-like G-proteins, MglA is a nucleotide (GTP)-dependent molecular switch protein, cycling between active (GTP-bound) and inactive (GDP-bound) states [26]. During motility, MglA-GTP localizes essentially at the leading cell pole and activates both T4P and the Agl/Glt system. The exact activation mechanism is unknown but may involve direct interactions with FrzS and AglZ [27]. The MglA GTP-hydrolysis activity is intrinsically low and is assisted *in vivo* by MglB, a GTPase-Activating Protein [16], [17]. MglB is a spatial regulator of MglA and localizes at the opposite lagging cell pole to inhibit MglA binding at that pole [16], [17]. Therefore, MglA and MglB form a polarity axis that can be inverted by the synchronous pole-to-pole switching of MglA and MglB, thus provoking a reversal [16], [17], [28]. Switching of MglAB is a regulated process and involves the signaling activity of the Frz signal transduction pathway, a chemosensory-like apparatus [16], [17], [28]. However, how Frz regulates the MglAB switch at the molecular level remains unknown. In summary, *Myxococcus* reversals are provoked by switching the activity of the motility systems (A and S) to the opposite cell pole which is under the control of MglA and the Frz signal transduction pathway.

In this study, we investigated how MglA localizes to the cell poles. We found that the polar localization of MglA requires RomR. Previously, it was shown that RomR, an essential A-motility protein, localizes to the cell poles in a Frz-controlled bipolar asymmetric pattern where it accumulates mostly at the lagging cell pole [29]. Since RomR contains a response regulator domain, its phosphorylation by the Frz kinase (FrzE) may directly contribute to A-motility regulation [29]. Revisiting the role of RomR, we found that RomR functions both for A- and S-motility and acts upstream from MglA, recruiting it to the cell pole. The results further show that the polarity axis builds from the formation of RomR-MglA and RomR-MglB complexes, leading to robust asymmetric protein localization at the poles. Finally, the evidence suggests that RomR may constitute a link between the Frz and the MglAB polarity control systems.

## Results

### Analysis of the switch motility protein localization interdependence network

**Figure 1:**
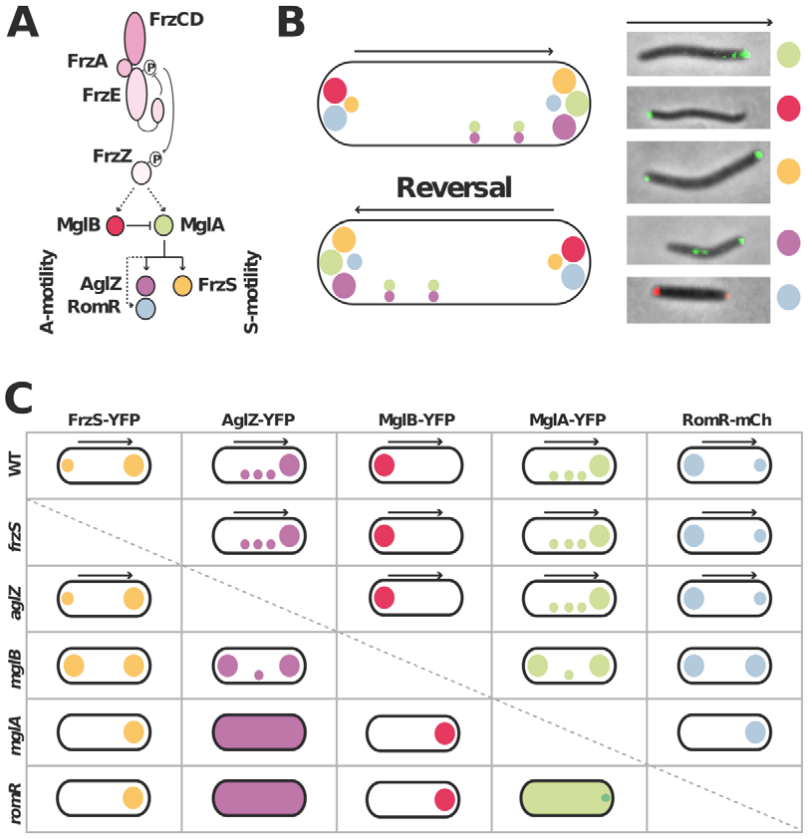
Analysis of motility protein localization interdependence network. (A) Schematic of the regulation cascade compiled from previous works. The essential signaling Frz components and established phosphate flow are shown. Plain arrows indicate established interactions and dotted arrows indicate suspected interactions. The protein color code applies throughout the manuscript. (B) Motility protein dynamics during the reversal cycle. The cartoon (left) is compiled from time-lapse studies of functional YFP fusions to MglA, MglB, FrzS, AglZ and RomR-mCherry during movement (right). The asymmetry of MglA, MglB, RomR, AglZ and FrzS at the poles is illustrated by circles of different sizes. The localization of MglA and AglZ along internal fixed A-motility clusters is also shown. The arrows represent the direction of movement. (C) Summary of motility protein localization patterns in WT and single deletion mutants. Black arrows denote a correlation between dynamic protein localization and cell reversals. Bipolar symbol of different sizes reflect asymmetric polar localization patterns as opposed to symmetric polar localization. The presence of internal fixed clusters is symbolized by distributed dots for simplicity.

Figure 1A&B recapitulates the known localization pattern of the previously studied switch and motility proteins, MglA, MglB, FrzS, AglZ and RomR. Previous works suggested an ordered pathway where Frz activates MglAB pole-to-pole switching to switch the localization of downstream motility system specific regulators such as FrzS (S-motility), AglZ and RomR (A-motility) [16], [17], [27], [29]. To confirm these studies in a definitive manner and identify localization interdependencies between these proteins, we systematically analyzed the localization of functional YFP/mCherry (mCh) fusions to MglA, MglB, FrzS, AglZ and RomR ([16], Figure S1, S2, S3) in all single mutants (summarized in Figure 1C and S2, S3, S4, S5). Most of the results were consistent with previous reports and confirmed that MglA and MglB are required to establish a polarity axis for motility: in the *mglA* mutant, AglZ-YFP became diffuse and failed to accumulate both at the pole and at periodic sites; FrzS-GFP, RomR-mCh and MglB-YFP localized only to one cell pole (Figure 1C [16], [17], [27], [29]). In the *mglB* mutant, all four proteins MglA-YFP, FrzS-YFP, AglZ-YFP and RomR-mCh showed bipolar symmetrical patterns (Figure 1C, Figure S2 and [16], [17]). The absence of FrzS or AglZ did not affect the localization of any of the other four proteins and thus must be branched downstream from MglA and MglB to regulate S- and A-motility, respectively (Figure 1C, S3, S4 and [27]).

**Figure 2:**
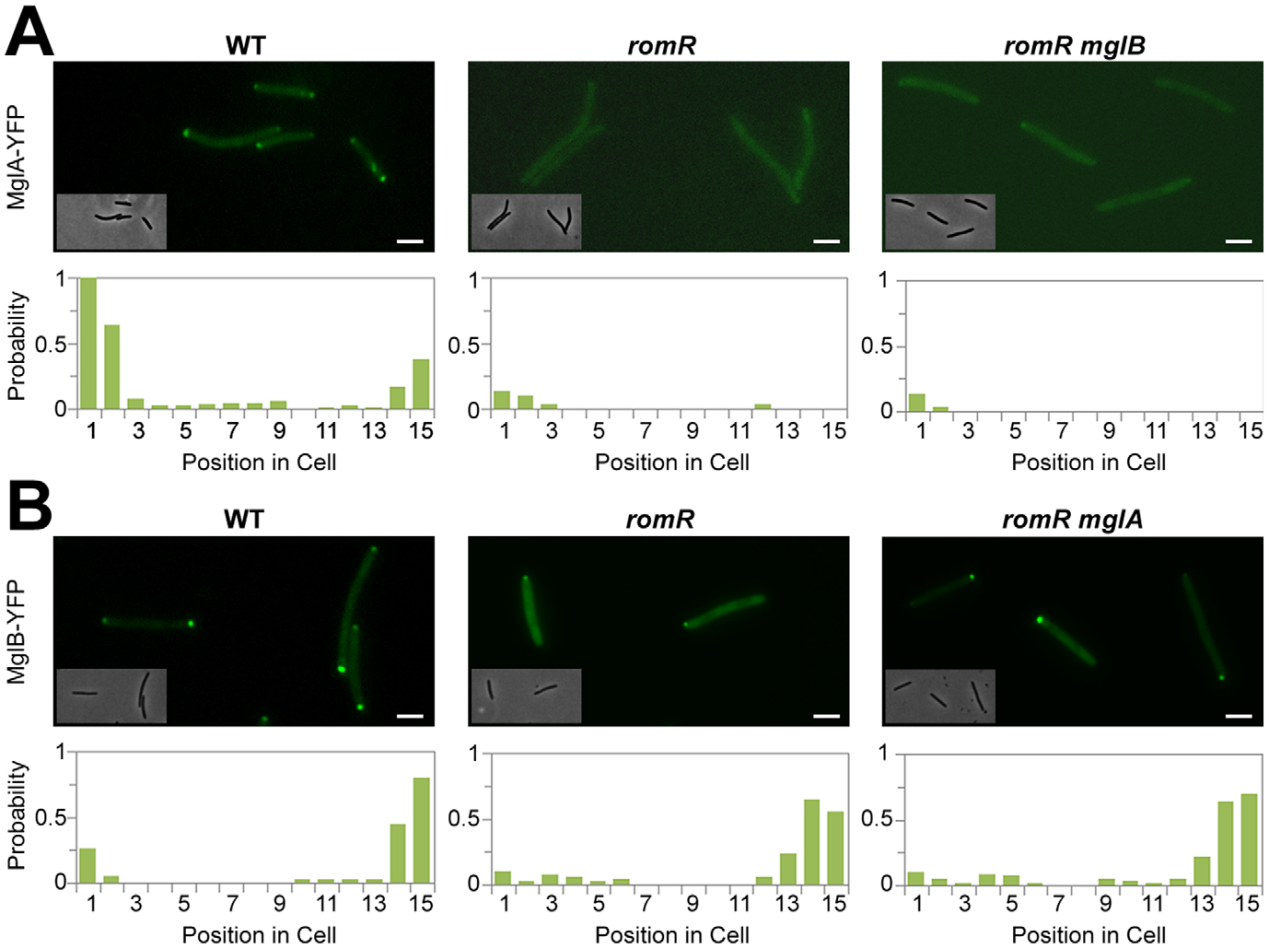
Localization of MglA and MglB in *romR* mutants. (A) Localization of MglA-YFP in WT, *romR* mutant and *romR mglB* mutant. (B) Localization of MglB-YFP in WT, *romR* mutant and *romR mglA* mutant. Insets: Corresponding phase contrast images. Histograms represent the occurrence of fluorescent clusters as a function of cell position divided in 15 equal segments (x-axis) for n = 100 cells of each strain (see methods for more details). Scale bar = 2 µm.

In a *romR* mutant, MglA-YFP and AglZ-YFP showed severe localization defects: AglZ-YFP was completely diffuse (Figure 1C and S5) and MglA-YFP localized in a largely diffuse pattern with only occasional minor polar foci forming in some cells (Figure 1C and 2A). The localizations of FrzS-YFP and MglB-YFP were less affected: both proteins localized to one cell pole but showed no dynamic pole-to-pole oscillations (Figure 1C, S5 and data not shown).

### RomR is required for MglA polar localization

In the *romR* mutant, the mis-localization of MglA cannot result from protein expression and/or stability defects because MglA accumulates to WT steady-state levels in the mutant as determined by Western blot analysis (Figure S6). Therefore, we further investigated the mechanism of RomR-dependent MglA polar localization. In absence of RomR, MglA mis-localization could either result from a direct defect in polar targeting or, more indirectly because MglB dynamics are affected, perturbing the spatial regulation of the MglB GAP activity. To discriminate between these possibilities, we tested whether deletion of *mglB* restores MglA-YFP localization. In WT cells, MglA-YFP mostly accumulates at the leading cell pole and gradually accumulates at the opposite cell pole between reversals [16], [17]. As a result, a fluorescence snapshot of MglA-YFP expressing cells shows a mix of cells with unipolar (60%) or with bipolar MglA-YFP clusters (40%, Figure 2A). Comparison of MglA-YFP in a *romR* and in a *romR mglB* mutant revealed that MglA-YFP localization is perturbed to comparable levels in the mutants: in both mutants, MglA-YFP was mostly diffuse and only accumulated as a minor unipolar cluster in less than 20% of the cells (Figure 2A). Thus, in a *romR* mutant, MglA-YFP mis-localization is largely independent from MglB and may result directly from the absence of RomR.

In the *romR* mutant, mis-localized MglA could interfere with MglB dynamics. To test this, we asked whether MglB-YFP dynamics are restored in a *romR mglA* double mutant. Consistent with its pole-to-pole dynamics MglB-YFP showed a mix of unipolar (75%) or bipolar patterns (25%) in WT cells (Figure 2B). In both *romR* and *romR mglA* mutants, MglB-YFP was strictly unipolar showing that MglB-YFP dynamics are not significantly restored in the *romR mglA* mutant. Therefore, the absence of MglB-YFP pole-to-pole dynamics in the *romR* mutant is not simply caused by MglA-mediated interferences but likely results from the global loss of function of MglA. Consistent with this, any mutant that lacks MglA (*mglA* mutant) or cannot localize it (*romR* mutant) is affected in MglB and other motility protein switching, for example FrzS (Figure 1C and S5).

### MglB is required for preferential accumulation of RomR at the lagging cell pole

**Figure 3:**
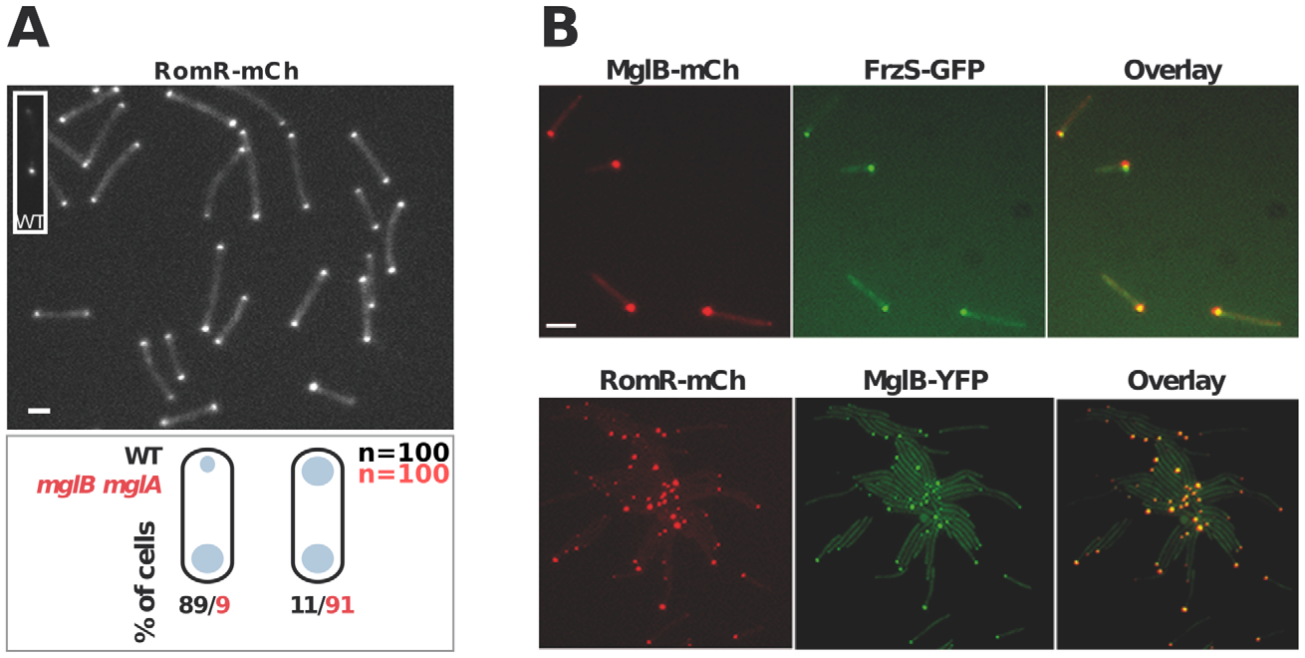
Polar localization of RomR is independent from MglAB but is regulated by MglB. (A) RomR localizes in a bipolar symmetrical pattern in absence of MglA and MglB. Inset: RomR localizes in a bipolar asymmetrical pattern in a WT cell. Cartoons: quantifications of the distinct localization patterns in either WT (black) or *mglB mglA* double mutant cells. Scale bar = 2 µm. (B) RomR, MglB and FrzS localizes to the same pole in absence of MglA. Protein localization in two-color *mglA* mutant strains expressing MglB-mCh/FrzS-GFP and RomR-mCh/MglB-YFP. Scale bar = 2 µm.

In WT cells, RomR localizes in a bipolar asymmetrical pattern and accumulates in larger amounts at the lagging cell pole (Figure 3A). How is this asymmetry generated and how does it relate to RomR function? RomR-mCh localized symmetrically to both cell poles in a double *mglAB* mutant, showing that RomR does not require MglA and MglB to bind to the cell poles (Figure 3A). Since RomR also localizes in a bipolar symmetrical pattern in the *mglB* mutant (Figure 1C) but only at one cell pole in the *mglA* mutant (Figure 1C and 3B), these results suggest that a RomR-MglB interaction captures RomR to the lagging cell pole. Consistent with this, dual-labeling experiments showed that RomR-mCh and MglB-YFP fusions localized to the same pole in the *mglA* mutant (Figure 3B). Of note, FrzS-GFP, which normally accumulates mostly at the leading cell pole [22], also co-localized with MglB-mCh (a functional MglB-mCherry fusion, [16]) and RomR-mCh in absence of MglA (Figure 3B, data not shown, see discussion). In total, these results suggest that RomR binds indiscriminately to the cell poles and that its asymmetric localization in WT cells stems from interactions with MglA at the leading cell pole and MglB at the lagging cell pole.

### RomR forms individual complexes with MglA and MglB

**Figure 4:**
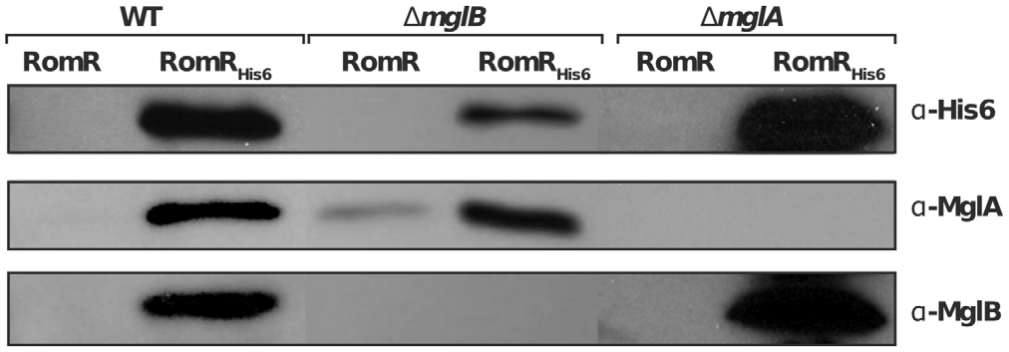
RomR interacts with both MglB and MglA independently. A functional RomR_His6_ was expressed from its endogenous promoter in the *romR* mutant respectively in otherwise WT, *mglA* and *mglB* mutant backgrounds and used in co-purification assays as described in the methods section. Co-purification specificity controls (RomR) are shown for each experiment and show that MglA and MglB are co-eluted only when RomR_His6_ is expressed (see methods).

We next tested whether the localization interdependencies between RomR, MglA and MglB stem from direct protein interactions at the cell poles. To test potential RomR-MglA and RomR-MglB interactions, we complemented the *romR* mutant strain with a construct allowing expression of RomR fused to a hexahistidine motif (His_6_) at its C-terminus, under the control of the *romR* promoter from a chromosome integration at the Mx8 phage attachment site [16]. Expression of RomR-His_6_ complemented the *romR* deletion showing that the tagged protein is fully functional (Figure S1). Interaction between RomR, MglA and MglB was then tested by affinity chromatography of *Myxococcus* soluble extracts on nickel columns (see Methods). Under these conditions, RomR readily co-purified with MglA and MglB, suggesting that MglA and MglB both interact with RomR (Figure 4). To further test whether RomR can form a complex with MglA and MglB independently, we conducted affinity chromatography of soluble extracts containing RomR-His_6_ expressed in *mglA* or *mglB* genetic backgrounds. Again, RomR-His_6_ co-purified with MglB and MglA in each case (Figure 4), suggesting that RomR forms independent complexes with MglA and MglB at cellular poles.

### RomR acts downstream from MglB and upstream from MglA to control motility

**Figure 5:**
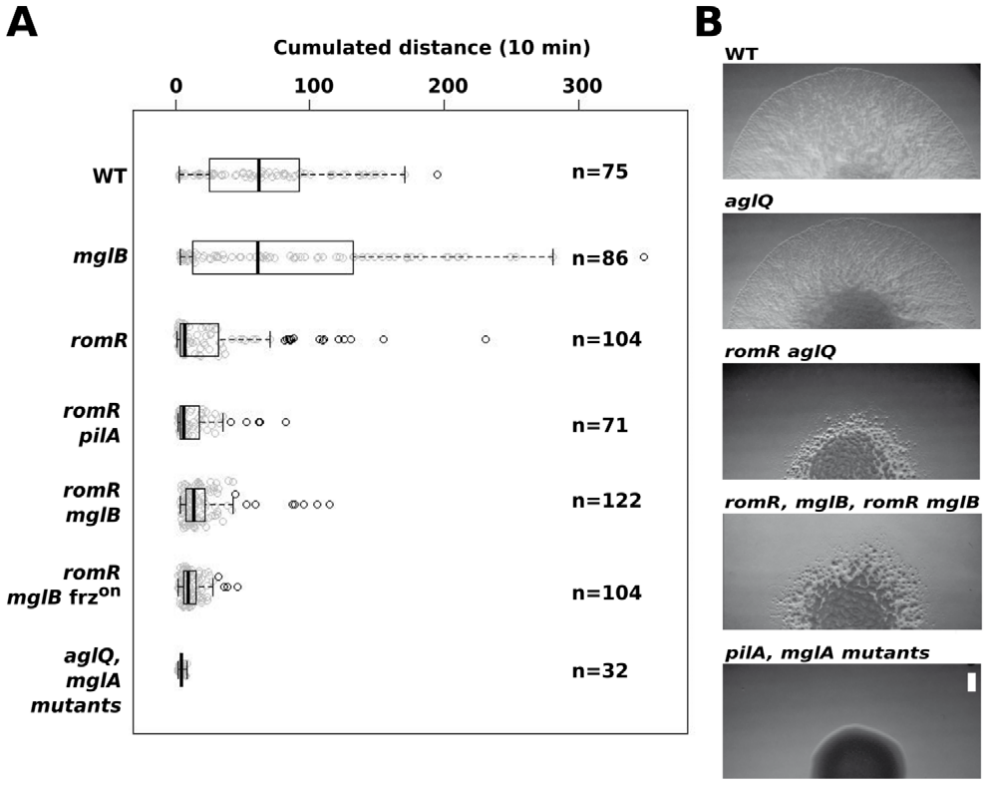
RomR acts downstream from MglB and upstream from MglA to control motility. (A) Single cell motility (A-motility) of the different strains on hard (1.5% w/w) agar. Shown are box plots of the measured cumulated distance traveled by isolated cells in 10 min for each strain. Gray circles represent each individual cell. The black bold vertical bar represents the average values for each strain and n indicates the number of cells that were tracked. Single *mglA*, *mglAB*, *romR mglA*, and *romR mglAB* mutant cells were completely non motile in this assay and were therefore collapsed in a single representative data set (*mglA* mutants). (B) RomR controls S-motility upstream from MglA. Soft (0.5% w/w) agar S-motility patterns of WT, *aglQ*, *romR, romR aglQ* and *pilA* mutant colonies. A representative micrograph is shown for mutant categories displaying identical swarming patterns: *romR, mglB*, *romR mglB* mutants and *pilA*, *mglA*, *mglAB*, *romR mglA*, *romR mglAB* mutants. Scale bar = 1 mm.

We next tested how a *romR* mutation affects A- and S-motility. We first analyzed the behavior of single A-motile cells on hard agar, a condition where S-motility is inefficient [30]. In this assay, *romR* mutant cells showed a severe motility defect and only exhibited limited back and forth movements (Figure 5A and Movie S1). This movement could be attributed to the A-motility system alone because it was still detectable in a *romR pilA* (S-) mutant but fully absent from a *romR aglQ* A-motility motor double mutant (Figure 5A and data not shown). The strong *romR* mutant A-motility defect is consistent with conclusions from Leonardy et al. [29] and the observed mis-localization of both MglA-YFP and AglZ-YFP in this mutant. To test the epistatic relationships between *romR* and *mglB*, we compared the motility defects of the *romR* mutant with the *mglB* and the *romR mglB* mutants. As previously reported, *mglB* mutant cells moved with a similar efficiency as WT cells but hyper-reversed (Figure 5A and Movie S2). In contrast, *romR mglB* mutant cells showed severely crippled motility, a motility phenotype that was comparable to the *romR* mutant phenotype (Figure 5A & Movie S1 & S3). Therefore, we conclude that RomR acts downstream from MglB to control A-motility. To determine whether *romR* acts upstream from *mglA*, we also compared the motility phenotypes of *mglA*, *mglA mglB*, *romR mglA* and *romR mglB mglA* mutants. All strains were completely non-motile and indistinguishable from the *mglA* mutant strain (and *aglQ* mutant strain), showing that MglA acts downstream from RomR and MglB (Figure 5A and Movie S4).

Since MglA is also important for S-motility, a *romR* mutation would also be expected to impact S-motility. This is further suggested by the fact that FrzS, an essential S-motility protein, fails to localize to both cell poles in the *romR* mutant (Figure 3B and S5). S-motility involves the movement of large cell groups and can be tested on soft agar (0.5% w/w), a substrate where A-motility is not effective [30]. In this assay and in WT cells, S-motility produces characteristic radial flares that emerge from a colony (Figure 5B). This pattern is unaltered in an *aglQ* mutant, confirming that A-motility is not active on soft agar (Figure 5B). On the contrary, a *romR* mutant displayed a severely defective swarm pattern (Figure 5B). This pattern results from a *bona fide* S-motility defect because a *romR aglQ* (A^−^) double mutant was defective to a similar extent (Figure 5B). In this highly qualitative assay, the comparison of the *romR* mutant with *mglB* and *romR mglB* mutants was not informative because all mutants displayed similar defective phenotypes (Figure 5B). However, clear epistatic relationships could be determined with *mglA* mutants: *mglA*, *mglAB*, *romR mglA* and *romR mglA mglB* mutants were all completely S^−^, showing that MglA acts downstream from RomR and MglB to control S-motility (Figure 5B). In total, the data strongly suggest that RomR acts downstream from MglB and upstream from MglA to both control A- and S-motility. The motility results are consistent with the localization results and a scenario where RomR acts as a polar targeting factor of MglA, the most downstream motility regulator. Finally, the correlation between motility phenotypes and localization defects suggests that polar localization of MglA is essential for its function.

### RomR regulates MglA localization downstream from Frz

**Figure 6:**
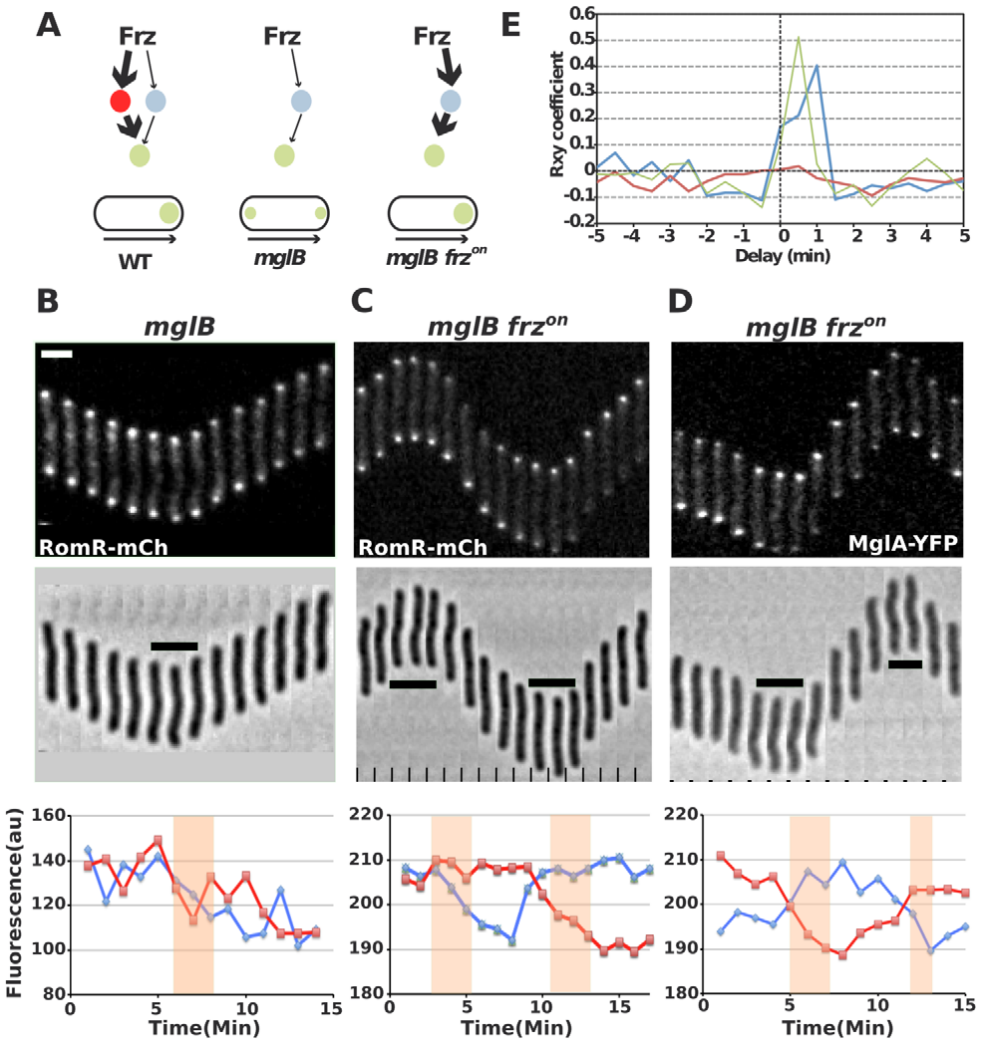
RomR regulates MglA localization in Frz-dependent and MglB-independent manners. (A) Frz-signaling trajectories to MglA and their predicted effect on MglA pole-to-pole dynamics. It is proposed that Frz regulates MglA through branched pathways involving MglB (major branch, thick arrows) and an additional regulator, possibly RomR (minor branch, thin arrows). In absence of MglB, FrzE signaling to RomR would be insufficient to support MglA dynamics. However, if Frz-signaling is enhanced (frz^on^ mutation, red thick arrows), sustained activation of the minor branch could restore MglA dynamics. (B) RomR-mCh dynamic localization in *mglB* mutant cells. Fluorescence micrographs and quantification of the corresponding fluorescence intensities (arbitrary units, au) of RomR-mCh at both poles over time are shown. Red line: initial lagging pole, blue line: initial leading pole. Reversals are indicated by thick black lines and orange rectangles on the micrograph and its corresponding graph. (C) RomR-mCh dynamic localization during reversals in *mglB frz^on^* cells. Legend reads as in (B). (D) MglA-YFP dynamic localization in *mglB frz^on^* cells. Legend reads as in (B). Scale bars = 2 µm. (E) Cross correlation between RomR-mCh and MglA-YFP pole-to-pole switching events and physical reversals in distinct genetic backgrounds. Red line = RomR-mCh in *mglB* background; Blue line = RomR-mCh in *mglB frz^on^* background; Green line = MglA-YFP in *mglB frz^on^* background.

The discovery that *Myxococcus* cell polarity arises from dynamic interactions between RomR, MglA and MglB raises the possibility that RomR acts immediately downstream from the FrzE histidine kinase to trigger the polarity switch. Several lines of evidence suggest that RomR is a possible Frz-output regulator. First, RomR is a modular protein containing both an N-terminal response regulator domain (RR) and a proline-rich C-terminal domain [29] and is thus a candidate substrate for FrzE. Consistent with this, RomR pole-to-pole switching is regulated by FrzE, which specifically requires the RomR receiver domain [29]. Additionally, expression of a RomR^D53E^ variant in which the conserved phosphorylatable Asp53 residue has been substituted by a Glutamate (a mutation expected to mimic a constitutively active state) bypassed the requirement for FrzE to trigger reversals [29]. Finally, there is evidence that Frz signaling is still transmitted to MglA in the absence of MglB, suggesting that Frz-signalling can be conveyed directly to MglA (Figure 6A, see [16] for details). Therefore, it is conceivable that RomR is part of this branch linking FrzE to MglA.

To test this possibility, we reasoned that a class of gain-of-function mutations in the *frzCD* receptor gene, the *frz* ^on^ mutations (frzCD^c^, [16]), may restore RomR and MglA pole-to-pole dynamics in absence of MglB. *frz^on^* mutations are thought to activate the Frz pathway constitutively [31], [32] and may thus hyper activate signals from FrzE to MglA via RomR (Figure 6A). In an *mglB* mutant, both MglA-YFP and RomR-mCh accumulated symmetrically at the cell poles and did not switch when cells reverse (Figure 6B, S2 and [16], [17]). Strikingly, RomR pole-to-pole switching was restored inversely in *frz^on^ mglB* mutant cells: RomR switched from leading cell pole-to leading cell pole, as opposed to WT cells where it switches from lagging cell pole to lagging cell pole (Figure 6C and [29]). As would be expected if RomR were the major MglA localization factor, MglA-YFP pole-to-pole switching was restored coincidently in *frz^on^ mglB* cells (Figure 6D). A cross correlation analysis done for 30 cells of each strain showed that significant Rxy scores (>0.4) near a 0–1 min time delay value were only obtained for RomR-mCh and MglA-YFP in *frz^on^ mglB* backgrounds, confirming that RomR-mCh and MglA-YFP polar fluorescence dynamics are correlated with physical reversals in *mglB frz^on^* mutant cells but not in *mglB* mutant cells (Figure 6E, see methods). To test whether RomR acts upstream from MglA in this regulation, we constructed an *mglB romR frz^on^* strain. *mglB romR frz^on^* mutant cells showed a *romR*-like motility defect (Figure 5A). Therefore, a *romR* mutation is epistatic over a *frz^on^* mutation, establishing that RomR acts downstream from Frz in the control of MglA dynamics. Finally, the results suggest that Frz signals do not require MglB to be conveyed to MglA.

## Discussion

### MglA defines the leading cell pole

What is the role of MglA polar localization? In all tested *romR* mutants, the severe mis-localization of MglA correlates with a profound motility defect, suggesting that polar MglA is essential to activate the A- and S-motility systems. It is possible that MglA regulates A- and S-motility in different ways. The S-motility proteins, FrzS and PilT, still localize to the pole in an *mglA* mutant (this work and [16], [17], [27], [29]). Importantly, FrzS and MglB/RomR, which are normally addressed to different poles, co-localize in *mglA* mutant cells. Therefore, MglA is not *per se* an S-motility protein polar localization factor but rather functions as an S-motility polar switching system. On the contrary, MglA is absolutely required for AglZ localization (this work and [27]). Therefore, the RomR-MglA-AglZ branch could determine localization of the A-motility machinery, while MglB and FrzS could be part of an A- (MglB) and S-motility (MglB and FrzS) polar switching pathway. In *Myxococcus*, MglA is thus required to differentiate a leading cell pole, similar to other Ras-family proteins defining the leading edge in chemotaxing eukaryotic cells [33]. How MglA does this exactly remains to be determined.

Unidentified polar cue(s) must exist to localize RomR, MglB and FrzS, which could be anchored by a common polar determinant or dedicated polar anchors. Motility machinery components themselves are likely not involved in this targeting because mutations in structural T4P or Agl/Glt proteins do not affect the reciprocal motility system [20], [34], [35] and any mutation that perturbs RomR or MglB localization would be expected to affect both systems. The polar cue(s) may be a general cell organizing structure, for example the cytokinetic machinery, lipid microdomains or membrane curvature itself [7], [8], [12], [14], [36].

### A three-protein interaction network creates a polarity axis

In a parallel study, Keilberg et al. obtained similar results and additionally suggested that RomR co-evolved with a subfamily of MglA-MglB systems [37]. More specifically, the results from both studies suggest strongly that *Myxococcus* dynamic polarity results from the action of three proteins, MglA, MglB and RomR. In this work, several lines of evidence suggest that a major function of RomR is to recruit MglA to the poles: (i) MglA localization depends on RomR but not *vice versa*. Additionally, MglA localization is not restored in a *romR mglB* double mutant, showing that RomR does not regulate MglA upstream from MglB. (ii), In *mglB* mutant cells, RomR and MglA localizations coincide in a bipolar symmetrical pattern. (iii), RomR and MglA switch poles coincidently in a *frz^on^ mglB* mutant. (iv), RomR is important for both A- and S-motility, which must be expected for any factor that regulates MglA. (v), RomR forms a complex with MglA in a co-purification assay. Last, Keilberg et al. [37] did not observe the restoration of MglA polar localization when YFP-MglA_Q82A_, an MglA GTP-locked variant [26], was expressed in the *romR* mutant, strongly suggesting that RomR is a direct MglA polar determinant. However, we cannot fully exclude that RomR also regulates the nucleotide-binding state of MglA (see below).

**Figure 7:**
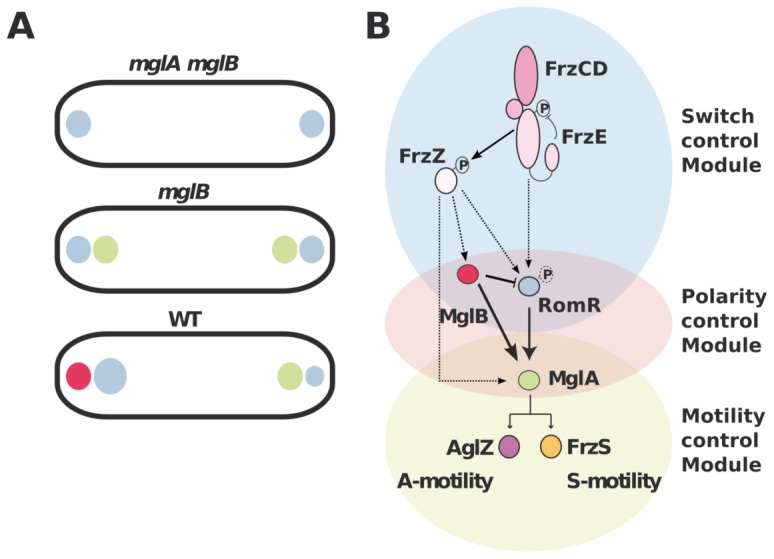
Polarity axis control by RomR, MglA, and MglB. (A) The interplay between RomR, MglA and MglB establish cell polarity. RomR is proposed to recognize the cell poles and direct efficient MglA localization to the poles. MglB polarizes the axis, which potentially results from two effects: the intrinsic MglB GAP activity and RomR attraction to the lagging cell pole. (B) Proposed regulatory cascade. Interactions established by this and previous works are represented by solid lines sum. Dotted arrows indicate possible connections between the upstream switch control system (Frz) and the polarity-control system (RomR-MglA-MglB), including RomR phosphorylation by FrzE and FrzZ-RomR, FrzZ-MglA and FrzZ-MglB interactions. MglB is proposed to act both positively to trigger MglA localization at the leading by its GAP activity (solid arrow) and negatively, by trapping RomR at the lagging cell pole (inhibitory symbol).

The MglA polar recruitment function of RomR was not initially obvious from its subcellular localization because RomR accumulates preferentially at the lagging pole where MglB is mostly present (Figure 1B & S1). In this study we show that MglB is directly responsible for this asymmetry and forms a complex with RomR at the back of the cells. The purpose of this regulation remains to be elucidated. We speculate that the MglB-RomR complex may further modulate accumulation of MglA–GTP at the leading pole as RomR becomes titrated by MglB. Therefore, MglB may exert two independent controls over MglA localization: directly with its GAP activity and indirectly, by trapping its localization factor RomR at the opposite cell pole. In the future, it could be interesting to determine if MglA and MglB compete for RomR binding to test this hypothesis. In summary, we propose that proper segregation of MglA and MglB at opposite cell poles requires a three-protein interaction network: first, RomR-MglA complexes direct MglA indiscriminately to the cell poles (Figure 7A). Second, MglB-MglA interactions repel MglA to the opposite cell pole and third, MglB-RomR interactions further modulate MglA levels at the leading cell pole. These concerted interactions thus create a robust polarity axis (Figure 7A).

### How does Frz trigger the polarity switch?

What is the genetic pathway leading to programmed cellular reversals? We propose a new genetic pathway that compiles this and previous studies (Figure 7B). In total, three main functional gene categories can be defined based on phenotypes: *frz* genes control the reversal frequency and thus trigger the polarity switch; *romR*, *mglA* and *mglB* form a cellular compass and thus affect both motility systems; *aglZ* and *frzS* are respectively specific to the A- and S-motility branch and thus connect the upstream genes to the motility apparatus. While MglA has been physically linked to AglZ and FrzS, how it is connected to the upstream Frz proteins remains unknown. The RomR protein itself is a strong candidate to connect FrzE kinase activity and MglA directly because RomR contains a response regulator (RR) domain and the intracellular dynamics of RomR are regulated by Frz-signaling (Figure 6 and [29]). Also, mutations in the conserved phosphorylatable Aspartate of the RomR receiver domain (D53N and D53E) are epistatic to a *frzE* mutation in the reversal pathway [29].

How is polarity switched dynamically by Frz-signaling? While the genetic data suggests that RomR is a likely FrzE output protein, biochemical evidence showing that RomR accepts phosphates directly from FrzE is still lacking. Other questions must also be resolved to model the switch mechanism. The interactions between the various players may be regulated at different levels and the proteins exist in several states, which may affect the protein localization interdependencies. For example, RomR phosphorylation may change its affinity for MglA and/or B, or even differentially to A and B. The formation of the RomR-MglA complex could also be dictated by the nucleotide state of MglA. Conversely, RomR may also regulate the nucleotide state of MglA, directly or indirectly by regulating MglB. As mentioned above, MglA and MglB could compete for RomR binding. Last, the Frz system may use several ouput proteins (Figure 7B). For example, the Frz pathway itself encodes two other receiver domain containing proteins, which are likely part of the regulatory pathway: the cognate FrzE receiver domain and the FrzZ protein [38], [39]. While the FrzE receiver domain may not be a direct Frz output domain and mostly regulate the phosphate flow in the system [38], [39], the phosphorylation of FrzZ is indispensable for the reversal switch [38], [39]. Therefore, the exact function of FrzZ and its exact connection with the RomR-MglAB system will also have to be clarified. In the future, a thorough biochemical analysis of Frz and RomR-MglAB interactions is required to elucidate the switch mechanism (Figure 7B).

### Conclusions

In bacteria, response-regulators are broadly used to target proteins to cellular poles. In *Caulobacter crescentus*, the cell-specific clearance of the master regulator CtrA licenses cell cycle progression [40]. In this regulation, CtrA is cleared specifically at the incipient stalked pole, by the combined action of two polar response regulator proteins [15], [41]. Response regulator proteins also regulate enzymes spatially. In another *Caulobacter* example, PleD an RR-containing protein localizes to the swarmer cell pole and orchestrates cell pole morphogenesis by activating local synthesis of the second messenger cyclic-di-GMP [42], [43]. Thus, RR domains implement subcellular organization by recruiting output proteins or domains to specific subcellular sites [44]. This versatility is further exemplified in *Myxococcus* where an RR protein (RomR) recruits a Ras-like G-protein (MglA) to the cell pole. This modular architecture is unique because it connects proto-typical prokaryotic and eukaryotic systems: a bacterial chemosensory-like pathway and a Ras-GAP system. In eukaryotes, Ras-GAP pairs generally act downstream from seven transmembrane receptors, the so-called G-Protein-coupled Receptors (GPCR), which sense ligands (ie cAMP) to activate Ras family proteins spatially [33]. In *Myxococcus*, where GPCRs are absent, the Frz pathway may therefore constitute a substitute activating module. This further illustrates how novel functions can arise from the conjunction of existing functional modules across the living kingdoms.

## Materials and Methods

### Bacterial strains, plasmids, growth conditions, and genetic constructs

See Table S1 for plasmids and Table S2 for strains and their mode of construction. Primer sequences and plasmid construction schemes are provided in Tables S3 and S4. *M. xanthus* strains were grown at 32°C in CYE rich media as previously described [45]. Plasmids were introduced in *M. xanthus* by electroporation. Mutants and transformants were obtained by homologous recombination based on a previously reported method [45]. Complementation, expression of the fusion and mutant proteins were either obtained by ectopic integration of the genes of interest at the Mx8-phage attachment site [16] under the control of their own promoter in appropriate deletion backgrounds or by expression from the endogenous locus (Table S2). Localization studies were performed using previously described FrzS-YFP, AglZ-YFP, MglB-mCherry/YFP functional fusions [16]. In the case of MglA-YFP, localization studies were performed in a merodiploid background where both MglA-YFP and MglA are expressed, a context where expression of MglA-YFP is not associated with detectable motility defects [16]. A functional RomR-mCh fusion protein was constructed for this study and expressed from the endogenous *romR* locus (Table S1, S2, S3, S4 and Figure S1).

For phenotypic assays, cells were spotted on CYE plates containing an agar concentration of 1.5% or 0.5% at a concentration of 4×10^9^ cfu ml^−1^, incubated at 32°C and photographed after 48 h with an Olympus SZ61 binocular or a Nikon Eclipse (model TE2000E) microscope.

### Western blotting

Western blotting was performed as previously described [16] with 1/10,000 dilutions of penta-His (QIAGEN), MglA or MglB antisera.

### Fluorescence imaging and fluorescence intensity measurements

Time-lapse experiments were performed as previously described [46]. Microscopic analysis was performed using an automated and inverted epifluorescence microscope TE2000-E-PFS (Nikon, France). The microscope is equipped with “The Perfect Focus System” (PFS) that automatically maintains focus so that the point of interest within a specimen is always kept in sharp focus at all times, in spite of any mechanical or thermal perturbations. Images were recorded with a CoolSNAP HQ 2 (Roper Scientific, Roper Scientific SARL, France) and a 40×/0.75 DLL “Plan-Apochro- mat” or a 100×/1.4 DLL objective. All fluorescence images were acquired with a minimal exposure time to minimize bleaching and phototoxicity effects.

Averaged fluorescent intensity profiles were computed as follows. A two-dimensional graph of the pixel intensities along the cell axis was first computed for each cell. The cell boundaries were then defined using a threshold value and the resulting restricted profile was spliced in 15 segments of equal length. The cluster probability distribution histograms were obtained as follows: the occurrence of a fluorescence cluster within a segment was detected by defining a minimal cluster intensity threshold value and the same threshold value was used for all conditions.

### Cell tracking and time-lapse fluorescence microscopy

Cell tracking was performed automatically using a previously described macro [46] under the METAMORPH software (Molecular devices); when appropriate, manual measurements were also performed to correct tracking errors with tools built into the software. Images were processed under both ImageJ 1.40 g (National Institute of Health, USA) and METAMORPH.

A measured single cell traveled distance represents the net distance travelled by a given cell, irrespective of its direction of movement. Therefore, reversals or any sort of back and forth movements are not accounted for in these measurements. The values were computed as the sum of the traveled distance by a given cell centroid in pixels during a 10 min reference time frame.

Statistical analysis of cell reversals was performed as previously described for 30 reversing cells of each tested strain [16]. The time-lapse movies are composed of 30 s time frames to avoid phototoxicity and photobleaching. Since a reversal takes on average 30 s between the initial pause and movement resumption, it is difficult to capture the exact time of the pause and fluorescence switching in our movies, creating noise in the analysis. Nevertheless, a reversal time was scored as soon as movement was detected in the opposite direction. MglA-YFP and RomR-mCh switching were scored when the maximum fluorescence was reached at the new leading pole. The cross-correlation coefficient (Rxy) between scored reversals and fluorescence pole-to-pole switchings for a time of delay (m) was calculated with the following equation:
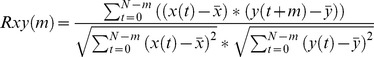
</disp-formula></xsl:text> Under these conditions and despite the low temporal resolution of the time lapse, significant Rxy values (the theoretical Rxy value for a perfect correlation is 1) could be obtained for a time delay near 0 value (±30 s), allowing to correlate fluorescence polar inversions (x(t)) and cellular reversals (y(t)) with confidence.


### Co-purification assays

Co-purification experiments were conducted by expressing a functional RomR_His6_ from its endogenous promoter at the Mx8 site in the *romR* mutant (which fully complemented the mutant, Figure S1), respectively in WT, *mglA* and *mglB* mutant backgrounds. As a control, co-purification specificity experiments were conducted in parallel using WT, *mglA* and *mglB* single mutants expressing un-tagged RomR to show that MglA and MglB were only recovered when RomR_His6_ is expressed. Co-purifications were conducted, after re-inoculating cells into 1L flasks to OD_600_ 0.4–0.8. The cells were collected by spinning at 5000 rpm for 20 min. The supernatant was discarded and the pellet was washed twice in wash buffer (NaH_2_PO_4_ 50 mM, NaCl 100 mM, MgCl_2_ 5 mM, pH 8.0) before being resuspended in 20 ml lysis buffer (NaH_2_PO_4_ 50 mM, NaCl 100 mM, MgCl_2_ 5 mM, 3 µl β-ME (Bio-Rad), 200 µl Protease Inhibitor Cocktail (PIC, Clontech), 10 µM GDP, 3 µl benzonase (Sigma), 20 mM Imidazole pH 8.0). The cells were then disrupted with a French press and spun down at 18,000 rpm, 4°C for 1 hour. The supernatants were then transferred into 50 ml Falcon tubes on ice and mixed with pre-equilibrated Nickel beads (Biorad). Beads and lysates were subsequently incubated at 4°C for 2 hours on a rotating wheel at 25 rpm. The bead-bound RomR complexes were then collected by low speed centrifugation (1 min at 2500 rpm) and washed three times in 50 ml lysis buffer. Elution was conducted by adding 100 µl of protein loading buffer (Leammli) directly to the beads and boiling at 100°C, for 10 min. Western blots were then conducted over 20 µL of the total elution volume under standard conditions to detect RomR_His6_, MglA and MglB.

## Supporting Information

Figure S1 Functional characterization of RomR-mCh and RomR_His6_. (A) RomR-mCh and RomR_His6_ are fully functional as judged by A and S motility assays. Strains expressing WT RomR, RomR-His6 or RomR-mCh were inoculated at the same time and incubated for 48 hrs on 0.5% agar or 1.5% agar plates to score for S and A-motility respectively. Micrographs were taken after 48 hrs incubations. Scale bars for 0.5% agar assay = 2 mm, for 1.5% motility assay = 20 µm. (B) RomR-mCh localizes asymmetrically at the lagging cell pole and switching is coupled with cell reversals in WT cells. Fluorescence micrographs and quantification of the corresponding fluorescence intensities of RomR-mCh at the poles over time are shown. Blue line: initial leading pole, red line: initial lagging pole. R: reversal.
(TIF)

Figure S2 Localization of MglA and RomR in an *mglB* mutant. (A) MglA-YFP in the *mglB* mutant. Fluorescence and micrographs and corresponding phase contrast overlaid images are shown. Scale bar = 2 µm. (B) RomR-mCh in the *mglB* mutant. Legend reads as in (A).
(TIF)

Figure S3 Localization and dynamics of FrzS, RomR and MglB in an *aglZ* mutant. (A) FrzS-YFP (Green) and RomR-mCh (Red) dynamics in absence of AglZ. A two color strain is used to test the dynamic and opposite pole-pole switching of FrzS and RomR. Fluorescence time-lapse micrographs overlaid on the corresponding phase contrast images are shown. R: reversal. Scale bar = 2 µm. (B) localization of MglB-mCh in the *aglZ* mutant. Scale Bar = 2 µm.
(TIF)

Figure S4 Localization and dynamics of AglZ, MglA, MglB and RomR in a *frzS* mutant. Localization and dynamics of AglZ-YFP (A), MglA-YFP (B), MglB-YFP (C) and RomR-mCh (D) in a *frzS* mutant. Fluorescence time-lapse micrographs overlaid on the corresponding phase contrast images are shown. R: reversal. Scale bars = 2 µm.
(TIF)

Figure S5 Localization of FrzS and AglZ in a *romR* mutant. Insets: Localization of the respective proteins in WT cells shown for comparison. For all strains, cells showing a given localization pattern were counted: numbers corresponding to specific localization patterns are shown next to illustrative cartoons in black for WT and red for *romR* mutants. FrzS-YFP panel: Fluorescent and phase contrast images are overlaid to show unipolar localization in the *romR* mutant. Scale bar = 2 µm.
(TIF)

Figure S6 Steady-state levels of MglB and MglA in the *romR* mutant. MglA and MglB were detected with appropriate antibodies in western blots on equivalent amounts of total proteins. A non-specific cross-reactive species detected with the anti-MglA antibody serves as a loading control (load).
(TIF)

Table S1 Plasmids used in this study.
(DOCX)

Table S2 Myxococcus strains.
(DOCX)

Table S3 Primers.
(DOCX)

Table S4 Description of plasmid constructions.
(DOCX)

Movie S1 Live observation of *romR* mutant cells on hard agar. Micrographs were taken every 30 s for a total time of 20 min.
(AVI)

Movie S2 Live observation of *mglB* mutant cells on hard agar. Micrographs were taken every 30 s for a total time of 20 min.
(AVI)

Movie S3 Live observation of *romR mglB* double mutant cells on hard agar. Micrographs were taken every 30 s for a total time of 20 min.
(AVI)

Movie S4 Live observation of *romR mglB mglA* triple mutant cells on hard agar. Micrographs were taken every 30 s for a total time of 20 min.
(AVI)
