# Supplementary figures and images for: A Dynamic Response Regulator Protein Modulates G-Protein–Dependent Polarity in the Bacterium Myxococcus xanthus
Source: PLoS Genet. 2012 Aug 16;8(8):e1002872. doi: 10.1371/journal.pgen.1002872 (PMC3420945; doi:10.1371/journal.pgen.1002872)

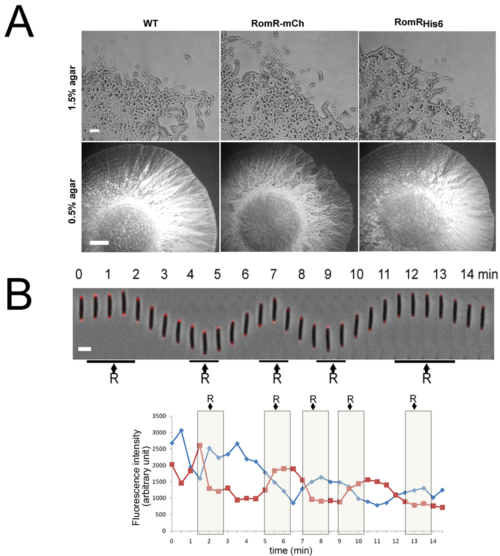

Supplement: Figure S1 — Functional characterization of RomR-mCh and RomRHis6. (A) RomR-mCh and RomRHis6 are fully functional as judged by A and S motility assays. Strains expressing WT RomR, RomR-His6 or RomR-mCh were inoculated at the same time and incubated for 48 hrs on 0.5% agar or 1.5% agar plates to score for S and A-motility respectively. Micrographs were taken after 48 hrs incubations. Scale bars for 0.5% agar assay = 2 mm, for 1.5% motility assay = 20 µm. (B) RomR-mCh localizes asymmetrically at the lagging cell pole and switching is coupled with cell reversals in WT cells. Fluorescence micrographs and quantification of the corresponding fluorescence intensities of RomR-mCh at the poles over time are shown. Blue line: initial leading pole, red line: initial lagging pole. R: reversal. (TIF) [file pgen.1002872.s001.tif]

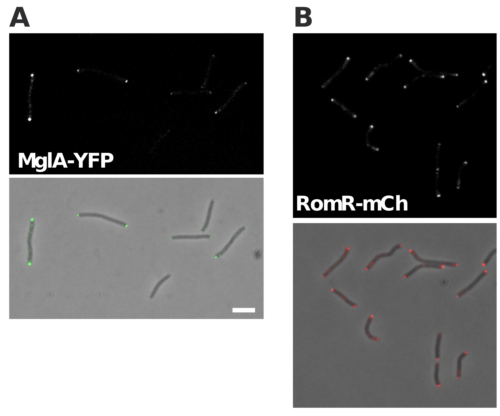

Supplement: Figure S2 — Localization of MglA and RomR in an mglB mutant. (A) MglA-YFP in the mglB mutant. Fluorescence and micrographs and corresponding phase contrast overlaid images are shown. Scale bar = 2 µm. (B) RomR-mCh in the mglB mutant. Legend reads as in (A). (TIF) [file pgen.1002872.s002.tif]

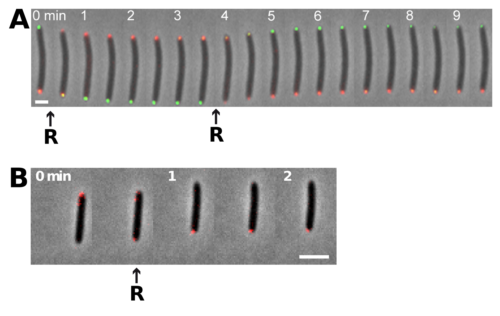

Supplement: Figure S3 — Localization and dynamics of FrzS, RomR and MglB in an aglZ mutant. (A) FrzS-YFP (Green) and RomR-mCh (Red) dynamics in absence of AglZ. A two color strain is used to test the dynamic and opposite pole-pole switching of FrzS and RomR. Fluorescence time-lapse micrographs overlaid on the corresponding phase contrast images are shown. R: reversal. Scale bar = 2 µm. (B) localization of MglB-mCh in the aglZ mutant. Scale Bar = 2 µm. (TIF) [file pgen.1002872.s003.tif]

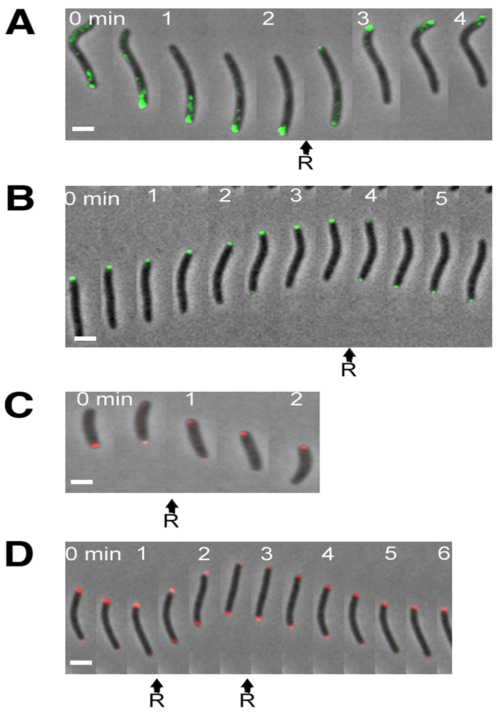

Supplement: Figure S4 — Localization and dynamics of AglZ, MglA, MglB and RomR in a frzS mutant. Localization and dynamics of AglZ-YFP (A), MglA-YFP (B), MglB-YFP (C) and RomR-mCh (D) in a frzS mutant. Fluorescence time-lapse micrographs overlaid on the corresponding phase contrast images are shown. R: reversal. Scale bars = 2 µm. (TIF) [file pgen.1002872.s004.tif]

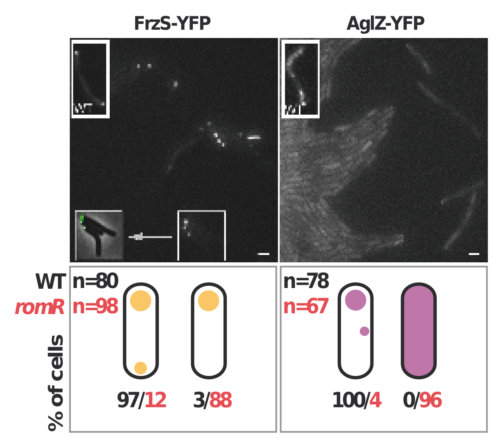

Supplement: Figure S5 — Localization of FrzS and AglZ in a romR mutant. Insets: Localization of the respective proteins in WT cells shown for comparison. For all strains, cells showing a given localization pattern were counted: numbers corresponding to specific localization patterns are shown next to illustrative cartoons in black for WT and red for romR mutants. FrzS-YFP panel: Fluorescent and phase contrast images are overlaid to show unipolar localization in the romR mutant. Scale bar = 2 µm. (TIF) [file pgen.1002872.s005.tif]

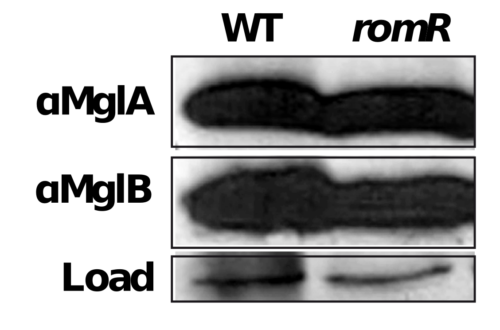

Supplement: Figure S6 — Steady-state levels of MglB and MglA in the romR mutant. MglA and MglB were detected with appropriate antibodies in western blots on equivalent amounts of total proteins. A non-specific cross-reactive species detected with the anti-MglA antibody serves as a loading control (load). (TIF) [file pgen.1002872.s006.tif]
