# Supplementary material for: A Dynamic Response Regulator Protein Modulates G-Protein–Dependent Polarity in the Bacterium Myxococcus xanthus
Source: PLoS Genet. 2012 Aug 16;8(8):e1002872. doi: 10.1371/journal.pgen.1002872 (PMC3420945; doi:10.1371/journal.pgen.1002872)
Supplement: Table S1 — Plasmids used in this study. (DOCX) [file pgen.1002872.s007.docx]

| Table S1. Plasmids |  |  |
| --- | --- | --- |
| Name | Description | Source |
| pBJ114  pSWU19  pSWU30  pEFrzSY  pBJAglZY  pBJmglAYR  pBJDmglB  pBJDmglBA  pSWU19mglB  pSWU19mglBY  pSWU30mglAY  pSWU30mglBC  pBJDaglQ  pBJDromR  pBJromRC  pSWU30-romR_His6_ | Used to create deletions, *galK*, Km^R^  Kan^R^ used to integrate genes ectopically at Mx8_att_  Tet^R^ used to integrate genes ectopically at Mx8_att_  pEYFPN1 with a cassette allowing construction of the *frzS-yfp* chimeric gene  pBJ114 with a cassette allowing construction of the *aglZ-yfp* chimeric gene  pBJ114 with a cassette allowing construction of the *mglA-yfp* chimeric gene  pBJ114 with a deletion cassette for *mglB*  pBJ114 with a deletion cassette for *mglBA*  pSWU19 allowing expression of *mglB* from its own promoter at Mx8_att_  pSWU19 allowing expression of *mglB-yfp* from its own promoter at Mx8_att_  pSWU30 allowing expression of *mglA-yfp* from its own promoter at Mx8_att_  pSWU30 allowing expression of *mglB-mCh* from its own promoter at Mx8_att_  pBJ114 with a deletion cassette for *aglQ*  pBJ114 with a deletion cassette for *romR*  pBJ114 allowing expression of *romR-mCh* from endogenous locus  pSWU30 allowing expression of *romR_His6_* from its own promoter at Mx8_att_ | [46]  L. Søgaard-Andersen  L. Søgaard-Andersen  [16]  [25]  [27]  [16]  [16]  [16]  [16]  [16]  [16]  [21]  This study  This study  This study |
