# Supplementary material for: A Dynamic Response Regulator Protein Modulates G-Protein–Dependent Polarity in the Bacterium Myxococcus xanthus
Source: PLoS Genet. 2012 Aug 16;8(8):e1002872. doi: 10.1371/journal.pgen.1002872 (PMC3420945; doi:10.1371/journal.pgen.1002872)
Supplement: Table S2 — Myxococcus strains. (DOCX) [file pgen.1002872.s008.docx]

| Table S2. *Myxococcus* strains | | | |
| --- | --- | --- | --- |
| Strain | Construction | Source | Genotype^a^ |
| DZ2  TM7  DZ4335  TM9  TM4  TM17  TM41  TM13  TM155  TM158  DZ4038  TM199  TM184  TM302  TM181  TM223  TM224  TM420  TM254  TM398  TM399  TM400  TM757  TM414  TM415  TM416  TM426  TM481  TM437  TM438  TM443  TM444  TM417  TM457  TM458  TM461  TM421  TM684  TM442  TM227  TM483  TM500  TM511  TM543  TM419  TM482  TM512  TM542  TM531  TM635  TM460 | Wild type  DZ2 *ΩaglZ frzS-gfp*  DZ2 Δ*frzS*  DZ2 *aglZ-YFP*  DZ2 Ω*frzE* *frzS-gfp*  DZ2 *mglA-yfp*  DZ2 Δ*mglA*  DZ2 Δ*mglA* *frzS-gfp*  DZ2 Δ*mglB*  DZ2 Δ*mglBA* (pBJDmglBA)  DZ2 *frz^on^*  DZ4038 *ΔmglB*  TM17 *mx8att::mglA*  TM184 *mglB-mCh*  TM155 *mx8att*::*mglA*-*yfp*  TM155 *frzS-yfp*  TM155 *aglZ-yfp*  TM155 *romR-mCh* (pBJromRC)  DZ2 *ΔromR* (pBJDromR)  TM254 *aglZ-yfp*  TM254 *frzS-yfp* (pEFrzSY)  TM254 *mglA-yfp* (pSWU30mglAY)  TM417 *mglB-yfp* (pSWU30mglBY)  TM41 *mglB-mCh* (pSWU30mglBC)  TM41 *romR-mCh* (pBJromRC)  TM13 *mglB-mCh* (pSWU30mglBC)  TM13 *romR-mCh* (pBJromRC)  TM415 *mglB-yfp* (pSWU30mglBY)  DZ4335 *aglZ-yfp*  DZ4335 *mglB-mCh* (pSWU30mglBC)  DZ4335 *romR-mCh* (pBJromRC)  DZ4335 *mglA-yfp* (pSWU30mglAY)  TM254 *ΔmglB* (pBJDmglB)  TM417 *aglZ-yfp*  TM417 *frzS-yfp* (pEFrzSY)  TM417 *mglA-yfp* (pSWU19mglAY)  TM7 *romR-mCh* (pBJromRC)  TM7 *mglB-mCh* (pSWU30mglBC)  DZ2 *romR-mCh* (pBJromRC)  TM199 *mglA-yfp* (pSWU19mglAY)  TM420 *frzCD^c^*  TM254 *ΔmglBA* (pBJDmglBA)  TM500 *mglB-yfp* (pSWU30mglBY)  TM500 *mglB* (pSWU19mglB)  TM158 *romR-mCh* (pBJromRC)  TM254 *ΩpilA*  TM254 *romR-his6* (pSWU30-romRhis6)  TM417 *romR-his6* (pSWU30-romRhis6)  TM254 *Δ*aglQ (pBJDaglQ)  TM41 *romR-his6* (pSWU30-romRhis6)  TM417 frz^on^ | Laboratory collection  Laboratory collection  Laboratory collection  [25]  [22]  [27]  [27]  [27]  [16]  [16]  [45]  [16]  [16]  [16]  [16]  [16]  This work  This work  This work  This work  This work  This work  This work  This work  This work  This work  This work  This work  This work  This work  This work  This work  This work  This work  This work  This work  This work  This work  This work  This work  This work  This work  This work  This work  This work  This work  This work  This work  This work  This work  This work | WT  *ΩaglZ frzS-gfp*  *ΔfrzS*  *aglZ-yfp*  ΩfrzE *frzS-gfp*  *mglA-yfp*  *ΔmglA*  *ΔmglA frzSGFP*  *ΔmglB*  *ΔmglBA*  *frz^on^*  *ΔmglB frz^on^*  *mglA-yfp mglA*  *mglA-yfp mglA mglB-mCh*  *ΔmglB mglA-yfp mglA*  *ΔmglB frzS-yfp*  *ΔmglB aglZ-yfp*  *ΔmglB romR-mCh*  *ΔromR*  *ΔromR aglZ-yfp*  *ΔromR frzS-yfp*  *ΔromR mglA-yfp mglA*  *ΔromR ΔmglB mglB-yfp*  *ΔmglA mglB-mCh*  *ΔmglA romR-mCh*  *ΔmglA frzSGFP mglB-mCh*  *ΔmglA frzSGFP romR-mCh*  *ΔmglA romR-mCh mglB-yfp*  *ΔfrzS aglZ-yfp*  *ΔfrzS mglB-mCh*  *ΔfrzS romR-mCh*  *ΔfrzS mglA-yfp mglA*  *ΔromR ΔmglB*  *ΔromR ΔmglB aglZ-yfp*  *ΔromR ΔmglB frzS-yfp*  *ΔromR ΔmglB mglA-yfp mglA*  *ΩaglZ frzS-gfp romR-mCh*  *ΩaglZ frzS-gfp mglB-mCh*  *romR-mCh*  *ΔmglB frz^on^ mglA-yfp mglA*  *ΔmglB frz^on^ romR-mCh*  *ΔromR ΔmglBA*  *ΔromR ΔmglBA mglB-yfp*  *ΔromR ΔmglA*  *ΔmglBA romR-mCh*  *ΔromR ΩpilA*  *ΔromR romR-_his6_*  *ΔromR ΔmglB romR-_his6_*  *ΔromR ΔaglQ*  *ΔmglA romR-_his6_*  *ΔromR ΔmglB frz^on^* |

^a^ A gene is italicized when present and preceded by the symbol Δ when deleted or the symbol Ω when interrupted by gene insertion.
