# Supplementary material for: A Dynamic Response Regulator Protein Modulates G-Protein–Dependent Polarity in the Bacterium Myxococcus xanthus
Source: PLoS Genet. 2012 Aug 16;8(8):e1002872. doi: 10.1371/journal.pgen.1002872 (PMC3420945; doi:10.1371/journal.pgen.1002872)
Supplement: Table S3 — Primers. (DOCX) [file pgen.1002872.s009.docx]

| Table S3. Primers | |  |
| --- | --- | --- |
| Plasmids | Name | Sequences of primers (5’---3’) |
| pBJDromR | DRomR2-1F  DRomR2-1R DRomR2-2F  DRomR2-2R | GGAATTCGATTCGCGCCATCTCCCATC GGGGTACCTTGACTCGCGGCAGTCTGAC  GGGGTACCCCGTAACCTCCTGACCTTTG GCTCTAGAACTTGCCGGTCTCCTTCGTC |
| pBJromRC | RmCherry-1F RmCherry-1R  RmCherry-2F  RmCherry-2R | CCCAAGCTTGAAGCGTTCGACGAGAATCG  GCCCGGCGCGCCAGAGCTCGAGCCAGAGTGCTGGGTCTCTCGGTCCT TCTGGCGCGCCGGGCATGGTGAGCAAGGGCGAGGA  GGAATTCTTACTTGTACAGCTCGTCCA |
| pSWU30-romR_his6_ | p30RH6-1F  p30RH6-1R | GCAGGTCGACTCTAGTTCCGCATGATCAGG  CCGGGGATCCTCTAGGCCAGGCGCCTCAGTGATGATGATGATGATGGTGCTGGGTCTCTCG |
