# Supplementary material for: A Dynamic Response Regulator Protein Modulates G-Protein–Dependent Polarity in the Bacterium Myxococcus xanthus
Source: PLoS Genet. 2012 Aug 16;8(8):e1002872. doi: 10.1371/journal.pgen.1002872 (PMC3420945; doi:10.1371/journal.pgen.1002872)
Supplement: Table S4 — Description of plasmid constructions. (DOCX) [file pgen.1002872.s010.docx]

| Table S4. Plasmid constructions | |
| --- | --- |
| Plasmid | Construction scheme^a^ |
| pBJDromR | Primer pairs DRomR2 1F/1R and DRomR2 2F/2R were used to amplify 1kb fragment upstream and downstream from the *romR* open-reading frame. Upstream and downstream fragments were digested with EcoRI/KpnI and KpnI/XbaI, respectively. Purified fragments were then ligated at the EcoRI and KpnI sites of pBJ114. The resulted construct was sequenced to ensure the absence of PCR-introduced mutations. |
| pBJromRC | Primers RmCherry-1F/1R were used to amplify *romR* from the DZ2 chromosome. Primers RmCherry-2F/2R were then used to amplify the fragment mCherry from the commercial plasmid pmCherry-1 (Clontech). Both fragments were fused by SOE PCR and cloned at the EcoRI and HindIII sites of pBJ114. |
| pSWU30-romR_his6_ | Primers p30RH6-1F /p30RH6-1R were used to amplify *romR* from the DZ2 chromosome. The purified PCR fragment was then ligated to XbaI digested pSWU30 with the in-fusion kit from Clontech. |

^a^ All plasmid inserts were sequenced to ensure the absence of PCR-introduced mutations.
